# Development of microsatellite markers and evaluation of the genetic diversity of the edible sea anemone *Paracondylactissinensis* (Cnidaria, Anthozoa) in China

**DOI:** 10.3897/BDJ.12.e134363

**Published:** 2024-09-25

**Authors:** Junyuan Li, Xuyi Yang, Zifeng Zhan, Juan Feng, Tinghui Xie, Yang Li

**Affiliations:** 1 College of Agriculture and Bioengineering, Taizhou Vocational College of Science and Technology, Taizhou, China College of Agriculture and Bioengineering, Taizhou Vocational College of Science and Technology Taizhou China; 2 Laboratory of Marine Organism Taxonomy and Phylogeny, Qingdao Key Laboratory of Marine Biodiversity and Conservation, Shandong Province Key Laboratory of Experimental Marine Biology, Institute of Oceanology, Chinese Academy of Sciences, Qingdao, China Laboratory of Marine Organism Taxonomy and Phylogeny, Qingdao Key Laboratory of Marine Biodiversity and Conservation, Shandong Province Key Laboratory of Experimental Marine Biology, Institute of Oceanology, Chinese Academy of Sciences Qingdao China

**Keywords:** Anthozoa, microsatellite DNA loci, population genetics, genetic diversity, ISO-seq

## Abstract

*Paracondylactissinensis* Carlgren, 1934 is a sea anemone with economic value in China. The wild population of *P.sinensis* has been shrinking due to overfishing and environmental pollution, which have caused price instability. In winter, the price of *P.sinensis* can reach 25 USD per kilogram. Up to now, there are no genetic markers developed for *P.sinensis*, preventing a further exploration of their population genetic diversity. In this study, the full-length transcriptome of *P.sinensis* was sequenced and microsatellite DNA markers (simple sequence repeats [SSRs]) were developed from those transcripts. A total of 52 primer pairs, which can amplify specific polymorphic bands in PCR experiments, were designed for the SSR markers. Genetic diversity and population genetics were analysed for *P.sinensis* populations collected from the coasts of Taizhou and Rizhao using six microsatellite DNA loci. While inbreeding was detected in both populations (F_is_ > 0), the overall number of alleles (Na = 11.3) and bottleneck analysis suggested that the genetic diversity of *P.sinensis* has not been greatly impacted. Clustering analyses using STRUCTURE, principal coordinate analysis and unweighted pair group method with arithmetic mean tree revealed that the Taizhou population diverged from the Rizhao population; however, the genetic differentiation between the populations was moderate. Human-mediated commercial activities may be the principal reasons for the gene flow between the populations. Our study provides the first evaluation of the genetic resources of wild *P.sinensis* populations in China, which can serve as a useful reference for future comparative studies on population genetics and may guide policy-makers in initiating strategies for germplasm conservation and artificial breeding.

## Introduction

The sea anemone *Paracondylactissinensis* Carlgren, 1934 (Cnidaria, Anthozoa) is distributed in the Indo-West Pacific Ocean and has been discovered mainly in countries such as Japan, China, India and Vietnam. *P.sinensis* lives in sandy beaches in intertidal or shallow subtidal zones. In China, *P.sinensis* has become an economic species and is widely accepted as a delectable delicacy, especially by local residents of Zhejiang Province (Li et al. 2023). However, industrial aquaculture for *P.sinensis* has not been established. The wild population of *P.sinensis* has decreased in recent years because of overfishing and other human activities. Accordingly, *P.sinensis* has become more expensive, with market prices fluctuating depending on the season (Li et al. 2023). Taizhou and Rizhao are cities in south China and north China where wild *P.sinensis* are typically caught for commercial use. However, no effective genetic markers are available for *P.sinensis*, which hinders access to the genetic diversity of this species or further investigations of its population genetics.

Up to now, only the mitochondrial genome and some mitochondrial barcodes are accessible in GenBank for *P.sinensis* (Li et al. 2023). Due to the slow evolutionary rates of the mitochondrial sequences in the anthozoans, mitochondrial DNA markers are limited in resolving genetic diversity at the population level for these organisms (Shearer et al. 2008). Microsatellite DNA markers are composed of simple sequence repeats (SSRs). As a result of their high degree of polymorphism, robust reproducibility, co-dominance and abundance in the genome, SSRs have become useful tools that are applicable in population genetics, parentage analysis, genetic linkage studies and molecular marker-assisted breeding (Park et al. 2009). With the development of high-throughput sequencing, SSRs can be easily and efficiently isolated from the genome or transcriptome databases of non-model organisms. SSRs for other species of sea anemones have been identified through computational searches of DNA databases derived from Illumina sequencing (Sherman et al. 2007, Gatins et al. 2016, Bocharova et al. 2018, Lane et al. 2020, Ryan et al. 2021). However, assemblies from short reads can introduce errors and the gene coding regions with useful application values are usually interrupted by introns or repetitive sequences. In this study, we used PacBio long reads (polished by Illumina short reads of RNA) (Tan et al. 2023, Teng et al. 2024) to generate a full-length transcriptome for *P.sinensis*. Using the obtained transcripts, SSRs were identified by repeat motif searches and a total of 52 polymorphic SSR markers were screened by PCR. Genetic diversity and population genetics were analysed for *P.sinensis* populations collected from the coasts of Taizhou and Rizhao. The results of this study provide the first information about the genetic resources for *P.sinensis* in China and serve as a reference for policy-makers to implement strategies associated with natural population conservation and breeding of improved genetic seeds.

## Materials and Methods

### Full-length transcriptome sequencing

One *Paracondylactissinensis* individual collected from the coast of Taizhou (121°39.5’E, 28°20.1’N) was selected for RNA extraction. The tentacle, body column and mesentery tissues were dissected and the TRIzol method (Invitrogen, USA) was used to isolate RNA. Contaminating DNA was removed using a TURBO DNA-free^TM^ Kit (Ambion, USA). The quantity and integrity of the extracted RNA were assayed with a Qubit fluorometer (Thermo Scientific, USA) and an Agilent Bioanalyzer 2100 (Agilent, USA), respectively. Equal amounts of RNA from different tissues were pooled to construct a library for PacBio sequencing. The ISO-Seq library was constructed following the protocols of the Preparing ISO-Seq^®^ libraries using SMRTbell^®^ prep kit 3.0 (PacBio, USA). SMRT sequencing was performed on a PacBio Sequel IIe sequencer (PacBio, USA). For Illumina sequencing, RNA from each tissue sample was used to construct sequencing libraries separately. Paired-end libraries with insert sizes of 300 bp were prepared from RNA using an NEBNext Poly(A) mRNA Magnetic Isolation Module of the NEBNext^®^ Ultra TM II RNA Library Prep Kit (NEB, USA). The libraries originating from the different tissues were sequenced on an Illumina NovaSeq 6000 sequencer (2 × 150 bp paired-end reads) (Illumina, USA). All sequencing services in this study were provided by Novogene Bioinformatics Technology Co., Ltd. (Tian Jin, China).

The long reads generated by the PacBio sequencer were processed according to the PacBio ISO-Seq pipeline (https://github.com/PacificBiosciences/IsoSeq/blob/master/isoseq-clustering.md). To polish the transcript consensus sequences clustered from PacBio long reads, both Illumina data (cleaned by Trimmomatic 0.36 (Bolger et al. 2014)) and PacBio data were used to improve the sequencing accuracy by NextPolish2 (Hu et al. 2024). Finally, CD-HIT v. 4.8.1 was used to remove redundancy in the transcripts with the following parameters: -c 0.90 -n 9 -T 0 -M 80,000 (Li and Godzik 2006). The coding regions of the transcripts were predicted by TransDecoder v.5.5.0 software (http://transdecoder.sourceforge.net/) and the resulting open reading frames (ORFs) were annotated by the NCBI NR protein database by the BLASTx tool v.2.2.31 + with an e value of less than 1×10 e^-5^.

### Microsatellite marker development

SSRs were detected with the MicroSatellite identification tool (MISA) software (Beier et al. 2017). The identified repeat types included mononucleotides, dinucleotides, trinucleotides, tetranucleotides, pentanucleotides and hexanucleotides. The primers used for each detected SSR were designed by the Batch Target Region Primer Design plugin of TBtools software (Chen et al. 2020). To select polymorphic and specific SSR markers, PCR experiments were performed on five *P.sinensis* individuals. The PCR mixture consisted of 15 to 30 ng of total genomic DNA, 2.0 µl of each primer (10 µM), 10.0 μl of 5× buffer, 4.0 µl of dNTPs (each 10 mM), 1.0 µl of PrimeSTAR HS DNA Polymerase (Takara, Japan) and PCR-grade water to a total volume of 50 µl. The PCR amplification programme was as follows: an initial denaturation step at 95°C for 5 minutes, followed by 25 cycles of denaturation at 95°C for 30 seconds, annealing starting at 65°C and decreasing by 1°C per cycle to 60°C for 30 seconds for each annealing step and extension at 72°C for 30 seconds. The reaction was terminated with a final extension step at 72°C for 5 minutes. The PCR products were detected using an SDS-PAGE gel stained by silver.

### Genetic diversity and population structure analyses

**Figure 1.**
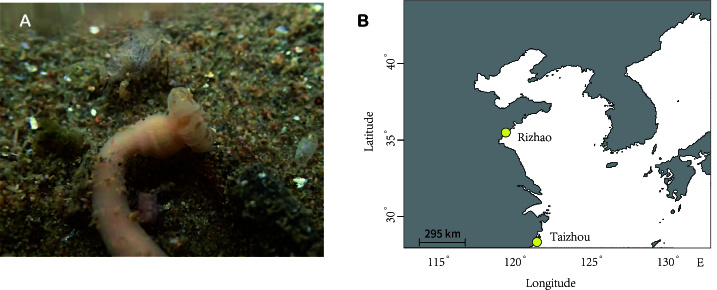
*In situ* picture of a *Paracondylactissinensis* settling on sand **(A)** and sampling sites of the populations analysed in this study **(B)**.

Sixteen individuals of *Paracondylactissinensis* were collected from the coasts of Taizhou, Zhejiang (121°39.5’E, 28°20.1’N) and Rizhao, Shandong (119°37.2’E, 35°30.3’N) respectively (Fig. 1Suppl. material 1) and all the 32 individuals were used for subsequent genetic analyses. Six primers were randomly selected from the microsatellite marker primer set developed in this study. Forward primers labelled with the fluorescent dye 6-FAM were synthesised. Combined with the corresponding unlabelled reverse primers, PCR was performed for all 32 *P.sinensis* individuals. The PCR system and programme were the same as those described above. The PCR products were then detected via capillary electrophoresis on a 3730XL DNA Analyzer (Applied Biosystems, USA). Allele sizes were visualised using GeneMapper software (Dash et al. 2020).

The genetic diversity indicators, including the number of (effective) alleles, Shannon index, observed heterozygosity, expected heterozygosity and inbreeding coefficient, were calculated by GenAlEx 6.5 (PEAKALL and SMOUSE 2005). BOTTLE NECK v. 1.2.02 (Piry et al. 1999) was utilised to detect recent reductions in effective population size. The two-phase model (TPM) is more appropriate for identifying bottleneck effects for microsatellite loci data (Cornuet and Luikart 1996), so it was chosen as the mutational model. The Wilcoxon test was employed to assess statistical significance. The Bayesian clustering approach, implemented using STRUCTURE v.2.3.4 (Pritchard et al. 2000), with 2 × 10^5^ Markov Chain Monte Carlo iterations and a burn-in period of 2 × 10^4^ was employed to infer population structure. The number of clusters (K) was evaluated from 1 to 10 and the results were analysed using StructureSelector (Li and Liu 2017) to determine the optimal K value. Cluster analysis was also performed using NTSYS-pc 2.10e software (Rohlf 1987). A dendrogram was created using the unweighted pair group method with arithmetic mean (UPGMA), which employs similarity matrices derived from the simple matching coefficient. Principal coordinate analysis (PCoA) was used to illustrate the multidimensional distributions of *P.sinensis* in a scatter plot. The source of genetic differentiation was analysed using an analysis of molecular variance (AMOVA) on the microsatellite dataset. The AMOVA analysis was conducted at three hierarchical levels: variances between the populations from Taizhou and Rizhao, variances amongst individuals within each population and variances within individuals. The fixation index (F_st_) and effective number of migrants (Nm) between the populations were calculated by GenAlEx 6.5 (PEAKALL and SMOUSE 2005).

## Results

### Statistics of transcriptome data

**Figure 2.**
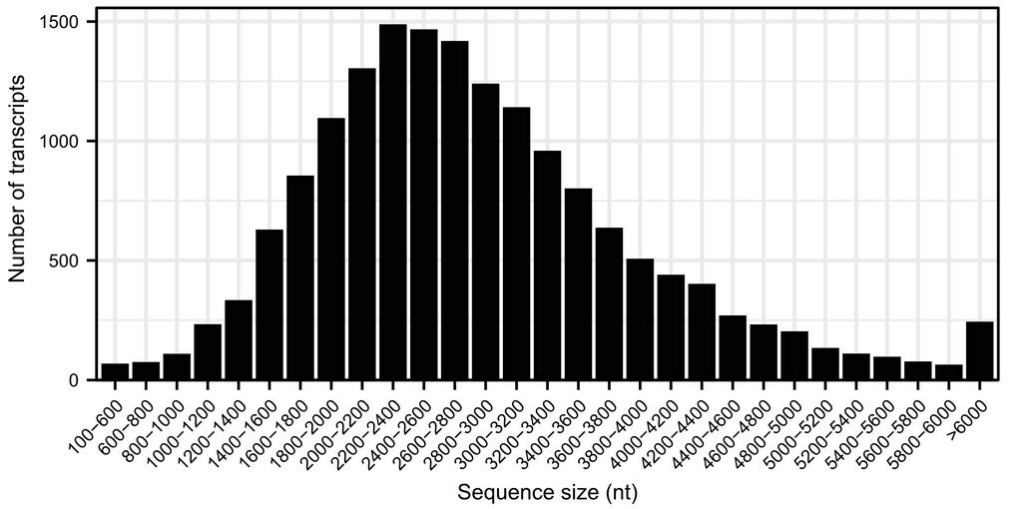
Length distribution of the transcripts of *Paracondylactissinensis*.

**Figure 3.**
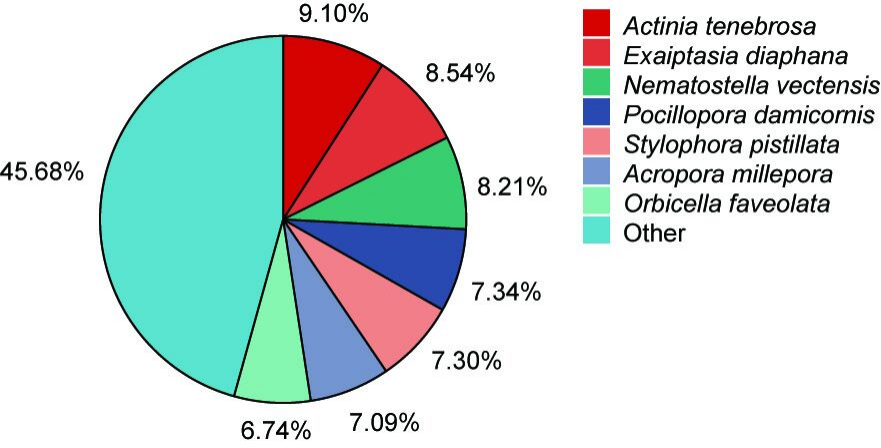
Species distribution for the annotation with the NR database.

A total of 1,112,498 polymerase reads were obtained using the PacBio Sequel II platform. After preprocessing, 142.47 Gb of subreads with an N50 length as long as 2,843 bp were obtained. The Illumina sequencer generated 8.68, 6.30 and 8.28 Gb of clean data for the tentacle, body column and mesentery tissues, respectively. The sequencing data produced in this study have been submitted to the National Center for Biotechnology Information Database under BioProject number PRJNA1086232. The clustering procedure for the PacBio long reads generated 54,259 transcripts, which included 54,219 high-quality transcripts and 40 low-quality transcripts. After polishing and redundant sequences removed, 16,490 non-redundant transcripts remained. The total length of these final transcripts was 47,089,935 bp, with an N50 length of 3,059 bp. The length distribution pattern of the transcripts indicated that most transcripts ranged from 2,000 bp to 3,000 bp in length (Fig. 2). Functional annotation revealed 14,829 transcripts with homologues in the NCBI NR protein database. Protein entries of sea anemones (*Actiniatenebrosa* 9.10%, *Exaiptasiadiaphana* 8.54% and *Nematostellavectensis* 8.21%) and corals (*Pocilloporadamicornis* 7.34%, *Stylophorapistillata* 7.30%, *Acroporamillepora* 7.09% and *Orbicellafaveolata* 6.74%) were most closely related (Fig. 3).

### Identification and screening of SSR markers

MISA software revealed 7,596 SSRs distributed within 4,867 transcripts. The distribution frequency of SSRs was 46.06% and the average interval between SSRs was 6.20 kb. The major SSR motifs were mononucleotide, trinucleotide and dinucleotide, accounting for 47.76%, 30.27% and 11.37% of the total number of repeats, respectively (Table 1). The number of repeats within each SSR ranged from 5 to 102. In general, for each type of repeat, as the number of repeats increased, the number of that type of repeat decreased accordingly (Table 1). To screen primers for SSRs, a total of 150 pairs of primers were randomly selected from the SSR primer sets designed with TBtools (Chen et al. 2020). After detection by electrophoresis with an SDS-PAGE gel, the primers amplifying unspecific or non-polymorphic bands were excluded (Suppl. material 2). Finally, 52 pairs of primers that generated specific and polymorphic amplified results were identified (Table 2). Six primers of the primer set were used for the subsequent assessment of genetic diversity (Table 2).

### Genetic diversity and population structure analysis

**Figure 4.**
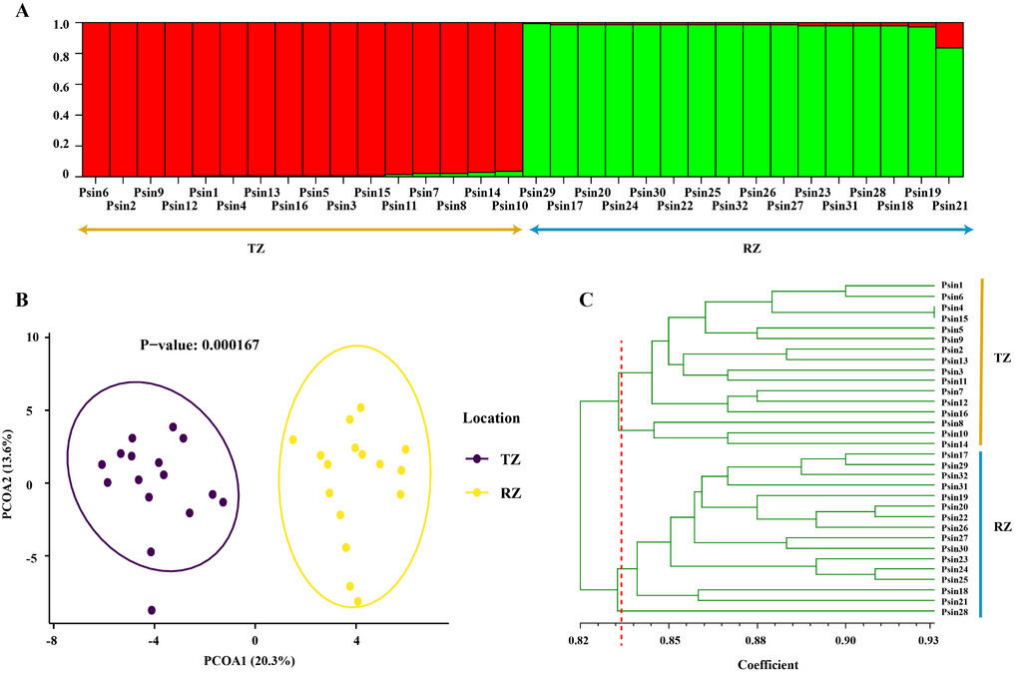
Population analyses of 32 *Paracondylactissinensis* individuals collected from Taizhou (16 individuals) and Rizhao (16). **(A)** Bayesian STRUCTURE clustering results; each colour represents the proportion of inferred ancestry from K (=2) ancestral populations, with each bar representing an individual sample; **(B)** Two-dimensional projection of the PCoA for 32 *P.sinensis* samples along the first two principal axes; **(C)** Relationships of *P.sinensis* populations based on genetic similarities derived from polymorphism patterns of SSR markers, displayed in a UPGMA dendrogram. TZ: Taizhou; RZ: Rizhao; Psin: *P.sinensis*.

The number of alleles (Na), number of effective alleles (Ne) and inbreeding coefficient (F_is_) of the *P.sinensis* population collected from Taizhou were 11.167, 7.713 and 0.085, respectively, which were relatively smaller than the corresponding values of the population collected from Rizhao (11.500, 7.833 and 0.118). The Shannon index (I), observed heterozygosity (Ho) and expected heterozygosity (He) were slightly greater in the Taizhou population (2.157, 0.781 and 0.856) than in the Rizhao population (2.107, 0.740 and 0.838) (Table 3). In a bottleneck analysis, populations with a significant excess or deficiency of heterozygosity are considered to have undergone a recent genetic bottleneck. According to the TPM, the bottleneck analysis indicated no genetic bottleneck effect in either population (p values for the Wilcoxon tests for both populations greater than 0.05). STRUCTURE analysis of the data, based on six microsatellite loci, indicated that the most likely number of genetic clusters was K = 2 (Ln P (D) = -1024.3; variance Var [Ln P (D)] = 149.4; ΔK = 8.49). This result suggested the presence of two distinct genetic groups amongst the 32 *P.sinensis* individuals in the present study. Moreover, the populations from Taizhou and Rizhao displayed apparent spatial structuring, with the 16 individuals of the Taizhou population clustering into one genetic group and the 16 individuals of the Rizhao population forming another distinct group (Fig. 4A). PCoA based on the codominant genotypic distance matrix of the six microsatellite markers also revealed that the Taizhou population was separate from the Rizhao population. The first and second axes of the PCoA explained 20.3% and 13.6% of the total molecular variance, respectively and the p value for the differentiation level between the two populations was smaller than 0.01 (Fig. 4B). An additional confirmation of the genetic separation between the Taizhou and Rizhao populations was provided by a dendrogram constructed using UPGMA analysis, based on genetic similarity coefficients. The result also indicated that the Taizhou and Rizhao populations were two distinct genetic groups (Fig. 4C), which was consistent with the results of the STRUCTURE and PCoA analyses. AMOVA revealed significant genetic variance between the two populations (9%, p < 0.01), amongst individuals within population (12%, p < 0.01) and within individuals (78%, p < 0.01) (Table 4). A comparison between the Taizhou and Rizhao populations indicated that the genetic differentiation characterised by the F_st_ value was 0.068 and the Nm between the two populations was 3.419.

## Discussion

### Advantages of the SSRs developed in this study

With the development of high-throughput sequencing technology, single nucleotide polymorphism (SNP) markers based population genomics have gained widespread use in studies aimed at understanding genetic diversity within species (Díaz et al. 2021, Wang et al. 2021, Yirgu et al. 2023), considering their higher power and resolution compared to SSR markers (Zimmerman et al. 2020). However, for practical applications involving polymorphism detection, the genetic signals of SSRs can be easily and economically collected using gel-based methods (such as polyacrylamide gel electrophoresis or capillary electrophoresis), which are less resource-intensive compared to SNP detection (usually realised by approaches, such as SNP arrays or next-generation sequencing). Furthermore, the species transferability of SNP markers is typically lower than that of SSR markers, when the SSRs are derived from coding regions (Park et al. 2009). The SSR markers developed in this study were derived from transcriptome data that contained rich coding regions. The flanking sequences of these SSRs are more conserved than the flanking regions of SNPs or SSRs previously reported for other sea anemones (Sherman et al. 2007, Gatins et al. 2016, Bocharova et al. 2018, Chan et al. 2020, Lane et al. 2020, Ryan et al. 2021), as the latter two are based on DNA sequences with a great proportion of non-coding regions characterised by variable sequences. Therefore, the primers of SSR markers developed in this study can potentially be applied across closely-related species with greater success.

### Genetic diversity of *Paracondylactissinensis*

The number of alleles (Na) is a parameter that can reflect genetic diversity and adaptability to changing environmental conditions. However, the value of Na is subjected to the number of samples and detection methods. The value of Na increases with the use of larger sample sizes or more sensitive detection methods. In a study by Remy Gatins (Gatins et al. 2016), the genetic diversity of four species of wild sea anemones from the Indo-Pacific was investigated using SSR polymorphisms. Even with a larger sample size (13-40 individuals) and the same detection method used in our study (capillary electrophoresis), only the populations of *Stichodactylagigantea* in that study presented greater Na values (12.6-13.4) than those of *P.sinensis* in China (~ 11.3). The populations of the other three species (*Entacmaeaquadricolor*, *Heteractismagnifica* and *S.mertensii*) presented significantly lower values (3.3-6.69). The relatively high Na value in *P.sinensis* reflects relatively rich genetic resources, which can increase the resilience of *P.sinensis* to changing environments. The overall inbreeding coefficient (F_is_) of *P.sinensis* is 0.101 (> 0), indicating that heterozygote deficits (Ho < He) were observed. However, the fact that the Ho was lower than the He may not be an indicator of low genetic diversity in the population, given that the greater number of genotypes could compensate for the lower population heterozygosity (Beardmore et al. 1997). Additionally, the absence of bottleneck effects in our results corroborates that the *P.sinensis* population has not experienced a drastic reduction in heterozygosity. Overall, the Na, Ho and He of *P.sinensis* did not exhibit significant signs of a decrease in genetic diversity. Nevertheless, it remains necessary to take a cautious attitude by formulating and implementing conservative strategies for wild *P.sinensis* populations in China, given the increasing severity of anthropogenic impacts, including overfishing, marine pollution and habitat destruction. These human-induced pressures, if unaddressed, may precipitate an irreversible decline in heterozygosity (a much greater F_is_), with far-reaching consequences for the genetic resources of *P.sinensis*.

### Population structure and gene flow

The STRUCTURE, PCoA cluster and UPGMA tree analyses revealed that the populations from Taizhou and Rizhao were two distinct genetic groups (Fig. 4). The difference between the genetic pools of the two populations could be associated with the different environments of Taizhou and Rizhao, cities in southern and northern China, respectively. *P.sinensis* is accustomed to warm waters and they can be collected year-round along certain coasts of southern cities. However, in northern cities, *P.sinensis* emerges along the coast only in the summer. Selective pressures (such as low temperature and low food availability) may restrict population size in northern China, which further decreases genetic diversity (reflected by lower Shannon index, Ho and He in the Rizhao population) and aggravates F_is,_ as shown in the results of this study (Table 3). F_st_ is a measure of genetic differentiation between populations. The F_st_ value between the populations of Taizhou and Rizhao was 0.068, indicating minimal genetic differentiation (an F_st_ less than 0.05 is often considered negligible (Curnow and Wright 1979)). This low population divergence seemed to contradict the substantial geographical distance between Taizhou and Rizhao (more than 1000 km, Fig. 1). Theoretically, the effects of genetic isolation by distance are more evident in low-mobility species, as gene flow tends to homogenise populations in some geographical areas (Avise 2008, Ferreira et al. 2014). While rapid seasonal oceanic currents could facilitate the dispersal of *P.sinensis* planktonic larvae, thus reducing population differentiation, the sedentary nature of adult *P.sinensis* compared with other mobile aquatic organisms might limit this effect. A low level of genetic divergence between geographically distant populations has been observed in other marine species (Benzie and Williams 1992, Apolônio Silva de Oliveira et al. 2017), which may be caused by multiple factors, such as human-mediated dispersal, genetic drift and population size (Yue et al. 2010). For the *P.sinensis* populations in China, commercial activity could be the principal factor. In Taizhou and nearby cities, *P.sinensis* is sold perennially in the seafood market and seafood vendors ship *P.sinensis* to northern cities in the cold seasons when the supply of wild *P.sinensis* is low. In this way, the genetic resources of southern populations have been introduced to northern districts, thus homogenising the genetic resources of the two populations to some extent. The analysis in this study revealed that the Nm reached 3.419, indicating that the gene flow between the two populations was high (an Nm greater than 1 is considered sufficient to counteract genetic drift and maintain genetic similarity between populations (Larson et al. 1984)). This gene flow resulted in greater Na value in the Rizhao population than in the Taizhou population (Table 3). The STRUCTURE analysis suggested that the individuals in the Rizhao population tended to contain more genetic elements from the Taizhou population than vice versa (Fig. 4A, the red proportion in the bars representing the Rizhao population was slightly larger than the green parts of the bars in Taizhou group), also congruent with the hypothesis of human-mediated dispersal. According to the AMOVA, the genetic variation between the northern and southern populations was only 9%, which was smaller than the variances amongst individuals (12%) and within individuals (78%). The small variances between populations could be associated with gene flow. The observed high variation within individuals is also apparent in other species (Karnosky 1991), which suggests that the high genetic resources in *P.sinensis* potently serves as a genetic pool for selective breeding in the future.

## Conclusions

This study represents the first successful development of microsatellite markers for *Paracondylactissinensis*, an economically valuable sea anemone in China. By employing a combination of PacBio long-read and Illumina short-read sequencing technologies, we identified 52 polymorphic SSR markers and utilised six microsatellite loci to evaluate the genetic diversity of *P.sinensis* populations from Taizhou and Rizhao. Our analyses revealed mild genetic differentiation between the two populations, with evidence of gene flow likely facilitated by human-mediated commercial activities. The absence of significant bottleneck effects and the relatively high genetic diversity, indicated by the number of alleles and heterozygosity levels, suggest that the genetic resources of *P.sinensis* have not been critically compromised. However, the detected inbreeding coefficients highlight the need for conservation strategies to safeguard the genetic integrity of this species in the face of ongoing environmental pressures. This research provides a crucial genetic baseline that can provide information for future studies and conservation efforts, offering valuable insights into the genetic management and sustainable utilisation of *P.sinensis* in China.

## Data resources

The raw data of sequencing reads for the transcriptome reported in this paper have been submitted in the National Center for Biotechnology Information (NCBI) database: BioProject PRJNA1086232.

## Supplementary Material

B78141F2-7631-55C8-8FB8-F4E7848DCF4F 10.3897/BDJ.12.e134363.suppl1 Supplementary material 1 Table S1. Sampling information of Paracondylactissinensis used for genetic diversity and population structure analyses. Data type Table File: oo_1135818.xlsx https://binary.pensoft.net/file/1135818 Li Junyuan

1CFDC720-EF72-562C-80BD-77E1C2926CBB 10.3897/BDJ.12.e134363.suppl2 Supplementary material 2 SDS‒PAGE gel showing the electrophoresis results of three SSR primers. The results of primer 91 are specific and reveal polymorphisms amongst the five individuals. The results of primers 94 and 95 are non-polymorphic and unspecific, respectively. Data type Image File: oo_1111038.jpg https://binary.pensoft.net/file/1111038 Li Junyuan

## Figures and Tables

**Table 1.** The number and distribution frequency of different SSR types in *Paracondylactissinensis*.

Type of repeat	Repeat times							Total	Ratio/%	Distribution Frequency/%
	5	6	7	8	9	10	>10			
Mononucleotide	\	\	\	\	\	\	3628	3628	47.76	22.00
Dinucleotide	\	284	162	116	68	37	197	864	11.37	5.24
Trinucleotide	1119	471	286	139	96	55	133	2299	30.27	13.94
Tetranucleotide	184	89	46	17	24	18	95	473	6.23	2.87
Pentanucleotide	19	7	1	5	1	6	28	67	0.88	0.41
Hexanucleotide	51	44	27	16	16	12	99	265	3.49	1.61
Total	1373	895	522	293	205	128	4180	7596	100	46.06
Ratio/%	18.08	11.78	6.87	3.86	2.70	1.69	55.03			

**Table 2.** Screening of SSR primer paris with specific and polymorphic resultant bands validated by PCR.

Marker name	SSR motif	Forward primer (5'-3')	Reverse primer (5'-3')	Product size (bp)
SS1	(AG)10	CCCAGGGAGTTGCCATTCT	TCCCCAAATCTCCATCTGCT	280
SS2	(TG)11	GGCAAATCCCAGCTCCC	TGTCGGCAAATGTTTTGACAG	278
SS3	(GT)14	GCTGTCGCATTGCTTCAGT	GGGCACACGTGACAAGG	271
SS4	(TC)20	ACGCCTTCTATAGCTCGCG	GGCGACCAACAGATGCG	265
SS5*	(GA)20	AGCCGTAGTAGACCCCGT	GCTCTGACGTCACGAGC	258
SS6	(TG)37	TGCCCTGAGATCAGCCC	ACATTACAGCTGGTTGGAGG	257
SS7	(GT)12	GCGCCCCCACCTATACTG	GCATCTGCAGTAAGGCGT	253
SS8*	(GA)14	AACACGTCCCTATCGGAGT	AACGGTCGAAAGGGGTCC	245
SS9*	(TATG)26	CGCCACTCATGCTTGCC	ACACTTGGAAGACCTTTTGCT	279
SS10	(TGT)9	CCAAGCCACGAAATCCTTGG	TCCGAGTCCCTGGCTGTT	279
SS11	(TTG)10	TGACGATGCGTGCAAGGT	ACAGCGAACGTTGACTTCT	278
SS12	(CAA)10	ACGCAAGCAATCCGTGG	GGCGCTGCCTGGATGTT	277
SS13	(TAGA)14	GGCCCAAATACGCTAACCG	CGCGGAAACAGGGGTAGG	274
SS14	(CCA)11	GCTCAGCACTTCGTACACCT	TCATGAACCCTGCTCCATC	272
SS15*	(TCT)8	TCAACTTCTCGCCGGCAG	GGGGAGGAGGGAAGGGAG	271
SS16	(AAGG)31	AGGCTAAGGACTGTCGCAG	CCGTCGCAAAACGTCTGG	269
SS17	(TTG)8	GGCCATGTTGTGAGAGACCT	AGGGCCGGTGGGAATAG	261
SS18	(GAT)13	AGGACAAATGCCCACCGAG	GGTTTCCGCCGTCTTCC	258
SS19	(TTCT)13	AGCATGCGGTCTGTCGAC	GCATTCTGGTCTAGCTGGGG	256
SS20	(GAT)10	AAAAGGAAGCCTGGCAAG	TGAGGGTCCAGCCTTGACT	255
SS21	(AGG)8	TAAGCGGCGTCTGTGC	ACTCGCGAGGGCTTCTTG	254
SS22	(ATG)13	ATGTCATACGGCAAAATGGC	TGGGTGGCATTCTTCGGT	253
SS23	(TGG)8	TGGTGGGGAGGGTGATG	TCACACATGCCCTCTTGAC	252
SS24	(GACA)9	TCTTGTCCCGGCTGCATG	CCCGTGGCAGACATCCAT	251
SS25	(ATG)12	AGTTGGCAGGGCAAATAAC	TGCCTTGTCAAAAATGTCCG	244
SS26	(GCTT)8	CCGGTAGGAACTTCCCTGG	TAACTGTCAACCCGCGCG	242
SS27	(AAC)9	CACCTACACCGCAAGCC	CACGAAACGCAAGCAGC	240
SS28	(TTCC)8	AGCTCCGTCTTTCCTTTCCT	GCATTACCACGTAAAATGCG	235
SS29	(TCTT)8	AGCCGAAGAGCTCTGGGT	TGCTTGCTTGTTCAGTGTTGG	233
SS30	(AAC)9	TGCAACAGCAAACGGGAAG	TCACATCACTGGTGAGGC	226
SS31*	(ACA)9	GGCTTGCAACCCCTTCG	GCCAGTGCCTTTCCTTTC	222
SS32	(AGA)8	GTGGTGGGCAACATGGGT	GCGTCAGTCGTGCCACT	222
SS33*	(TTG)8	GCTCGACTCAGCTGCGT	TGACAGTAGACAGCTACCAC	219
SS34	(AAC)8	ACCCCTAACAACAGTGGGC	ATCTCGGGTCGCCAAACC	214
SS35	(CGT)9	CGGGGTGGCTATGCGATC	AGATCACTAAAGCTGCAGAC	197
SS36	(CAA)9	ACAGCAGGCACGTTTCC	AGGGTGGAGGTCGCATCT	185
SS37	(TTTC)15	AGGCATTAGGTCATTCGGACT	TTCGCACGGGGTCTTTCC	176
SS38	(TTG)8	TAAGCGCAAGGCCCAC	ACCTTGTGCACTGCTAGCT	174
SS39	(GTT)11	TGCCGAGTTTCACAGCG	GCGCGTCTTTGTTGTTGC	147
SS40	(ATG)8	TGAAAGTGCGCCCGAAGT	GGTGATGGTACGTCAGTCAGT	147
SS41	(AGA)8	CCGTCTGCTCTTGCCAG	TCACAACGTAACGGACAGC	109
SS42	(TCA)8	CGGATCGTCACGTCACC	AGCAACACCTTTGTTTTGTGT	108
SS43	(TTTAC)8	ATGACGGCCGAAACCACG	GGGAAAAGTTTGGTACTCGGT	272
SS44	(TTTTG)24	CGAGAACTCGTTTGTGTTGTT	AGCTGCTTCACTTTGGTCTT	262
SS45	(T)20	CCTGAGTCAGTTACGCAAAGG	AGCCATCAAAAGTTACAGGCT	229
SS46	(TGA)11	CCTCCAGGGATGAAGGCG	TTGGCCCGATGACAGGTC	127
SS47	(TC)20	TGCTTCACTCACCCATGGG	AGCCTCTCCTACTCACAGCT	129
SS48	(TG)32	ACCGACGCGTTGAAAGG	CGTTCTCACATCCAAAACGGT	149
SS49	(CT)15	GAAGTTGGCCCTAGCGC	TGGACCAAGGTTACTGGACAC	170
SS50	(TC)13	CCCTTCTCAGTGGTTGGC	ATCTCGCGGGAGGAGAGT	174
SS51	(TG)19	TGTGAGGATTTGGAGGTTTCG	GCATAGCAAAACCAGGCAC	184
SS52	(GAT)14	GCAACCATGGATGATACCGC	TGCTGTCTTCATCGTGGCT	187
Note: the marker name labelled with a star indicates its following usage in fluorescent primer synthesis and population genetic analysis.				

**Table 3.** Genetic diversity parameters for the populations of *Paracondylactissinensis* from Taizhou (TZ) and Rizhao (RZ).

Population	N	Na	Ne	I	Ho	He	F_is_
TZ	16	11.167	7.713	2.157	0.781	0.856	0.085
RZ	16	11.500	7.833	2.107	0.740	0.838	0.118
Mean	16	11.333	7.773	2.132	0.760	0.847	0.101
Note: N: number of sapmles; Na: number of different alleles; Ne: number of effective alleles; I: Shannon's information index; Ho: observed heterozygosity; He: expected heterozygosity; F_is_: inbreeding coefficient.							

**Table 4.** Analysis of molecular variance for the *Paracondylactissinensis* from Taizhou and Rizhao, based on six microsatellite loci data.

Source of variation	df	Sum of squares	Estimated variance	Percentage of variance
Between populaitons	1	11.719	0.273	9%
Amongst individuals within populations	30	89.594	0.353	12%
Within individuals	32	73.000	2.281	78%
Total	63	174.313	2.907	100%
