# Supplementary figures and images for: Development of microsatellite markers and evaluation of the genetic diversity of the edible sea anemone Paracondylactissinensis (Cnidaria, Anthozoa) in China
Source: Biodivers Data J. 2024 Sep 25;12:e134363. doi: 10.3897/BDJ.12.e134363 (PMC11447459; doi:10.3897/BDJ.12.e134363)

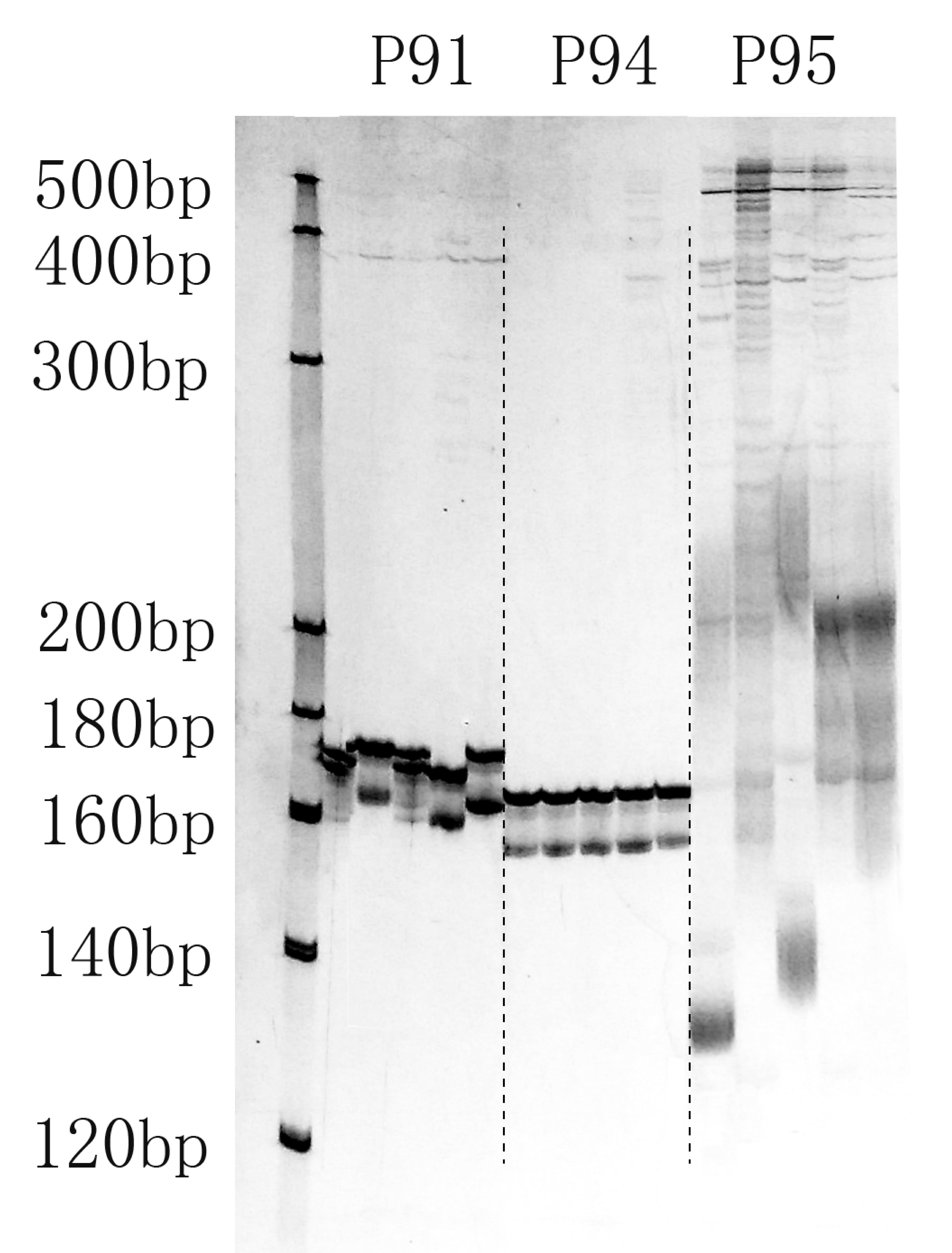

Supplement: Supplementary material 2 — SDS‒PAGE gel showing the electrophoresis results of three SSR primers. The results of primer 91 are specific and reveal polymorphisms amongst the five individuals. The results of primers 94 and 95 are non-polymorphic and unspecific, respectively. [file bdj-12-e134363-s002.jpg]
